# [^11^C]flumazenil Binding Is Increased in a Dose-Dependent Manner with Tiagabine-Induced Elevations in GABA Levels

**DOI:** 10.1371/journal.pone.0032443

**Published:** 2012-02-27

**Authors:** W. Gordon Frankle, Raymond Y. Cho, N. Scott Mason, Chi-Min Chen, Michael Himes, Christopher Walker, David A. Lewis, Chester A. Mathis, Rajesh Narendran

**Affiliations:** 1 Department of Psychiatry, University of Pittsburgh, Pittsburgh, Pennsylvania, United States of America; 2 Department of Radiology, University of Pittsburgh, Pittsburgh, Pennsylvania, United States of America; 3 Department of Neuroscience, University of Pittsburgh, Pittsburgh, Pennsylvania, United States of America; Chiba University Center for Forensic Mental Health, Japan

## Abstract

Evidence indicates that synchronization of cortical activity at gamma-band frequencies, mediated through GABA-A receptors, is important for perceptual/cognitive processes. To study GABA signaling in vivo, we recently used a novel positron emission tomography (PET) paradigm measuring the change in binding of the benzodiazepine (BDZ) site radiotracer [^11^C]flumazenil associated with increases in extracellular GABA induced via GABA membrane transporter (GAT1) blockade with tiagabine. GAT1 blockade resulted in significant increases in [^11^C]flumazenil binding potential (BPND) over baseline in the major functional domains of the cortex, consistent with preclinical studies showing that increased GABA levels enhance the affinity of GABA-A receptors for BDZ ligands. In the current study we sought to replicate our previous results and to further validate this approach by demonstrating that the magnitude of increase in [^11^C]flumazenil binding observed with PET is directly correlated with tiagabine dose. [^11^C]flumazenil distribution volume (VT) was measured in 18 healthy volunteers before and after GAT1 blockade with tiagabine. Two dose groups were studied (n = 9 per group; Group I: tiagabine 0.15 mg/kg; Group II: tiagabine 0.25 mg/kg). GAT1 blockade resulted in increases in mean (± SD) [^11^C]flumazenil VT in Group II in association cortices (6.8±0.8 mL g−1 vs. 7.3±0.4 mL g−1;p = 0.03), sensory cortices (6.7±0.8 mL g−1 vs. 7.3±0.5 mL g−1;p = 0.02) and limbic regions (5.2±0.6 mL g−1 vs. 5.7±0.3 mL g−1;p = 0.03). No change was observed at the low dose (Group I). Increased orbital frontal cortex binding of [^11^C]flumazenil in Group II correlated with the ability to entrain cortical networks (r = 0.67, p = 0.05) measured via EEG during a cognitive control task. These data provide a replication of our previous study demonstrating the ability to measure in vivo, with PET, acute shifts in extracellular GABA.

## Introduction

Accumulating evidence indicates that synchronization of cortical neuronal activity at gamma-band frequencies (30–80 Hz), mediated through GABA-A receptor transmission, is important for various types of perceptual [1], [2], [3] and cognitive processes [4], [5]. In order to assess the relationship between changes in gamma band power and extracellular GABA levels, we recently using a novel positron emission tomography (PET) brain-imaging paradigm to measure the in vivo binding of the benzodiazepine (BDZ) site specific radiotracer [^11^C]flumazenil [6] at baseline and in the context of elevated GABA levels induced via blockade of the GABA membrane transporter (GAT1) with tiagabine (Gabitril®) [7]. Preclinical work suggests that increased GABA levels enhance the affinity of GABA-A receptors for BDZ ligands via a conformational change (termed the ‘GABA-shift’) [8], [9], [10]; such an increase in affinity of GABA-A receptors should be detected as an increase in the binding of a GABA-A BDZ-receptor site-specific PET radioligand. In our study, GAT1 blockade resulted in significant increases in [^11^C]flumazenil binding potential (*BP*_ND_) over baseline in brain regions representing the major functional domains of the cerebral cortex and this increase strongly predicted (r = 0.85, p = 0.015) the ability to entrain cortical networks, measured via EEG gamma synchrony during a cognitive control task in these same subjects [7]. These findings are consistent with the results of experimental models [11], [12] as well as preclinical studies [13], [14], [15] suggesting that GABA-A receptor-mediated transmission is required for the induction of gamma network oscillations.

The aim of the current study was to replicate our previous results and to further validate the methods by demonstrating that the magnitude of increase in [^11^C]flumazenil binding observed with PET is directly correlated with the degree of GABA increase. The refinement and validation of the PET methodology described previously would provide a unique ability to measure changes in extracellular GABA levels in vivo; examine the relationship between GABA neurotransmission, oscillatory activity and cognition; and to explore differences between control and patient populations in the degree of extracellular GABA increase in response to a standardized level of GAT1 blockade. Moreover, if abnormalities in GABA transmission exist in psychiatric disorders as suggested by recent studies in schizophrenia [16] and major depression [17], this technique could be employed in the process of developing new pharmacologic compounds with the target of increasing cortical GABA levels. Eighteen healthy volunteers underwent two [^11^C]flumazenil PET scans on the same day, baseline and 60 minutes after administration of oral tiagabine; nine subjects received a dose of 0.15 mg/kg and nine subjects received a dose of 0.25 mg/kg. We hypothesized that the increase in [^11^C]flumazenil binding after the administration of tiagabine will occur in a dose-dependent manner.

## Materials and Methods

### Human Subjects

The study was approved by the Institutional Review Board of the University of Pittsburgh Medical Center. Eighteen healthy volunteers provided written, informed, consent and participated in this study in two dose groups (Group I: tiagabine 0.15 mg/kg, age 24±3 years, range 21 to 29, 4M/5F; Group II: tiagabine 0.25 mg/kg, age 30±11 years, range 19 to 48, 5M/4F; with these and subsequent values given as mean ± SD). The absence of pregnancy, medical, neurological and psychiatric history (including alcohol and drug abuse) was assessed by history, review of systems, physical examination, routine blood tests including pregnancy test, urine toxicology and EKG. Subjects provided written informed consent after receiving an explanation of the study.

### PET protocol

All subjects were studied twice with [^11^C]flumazenil on the same day. On the study day an arterial catheter was inserted in the radial artery, after completion of the Allen test and infiltration of the skin with lidocaine, for blood sampling and a venous catheter was inserted in a forearm vein for radiotracer injection. First, a baseline PET scan was performed. The baseline scan was followed by oral administration of tiagabine (Group I 0.15 mg/kg; Group II 0.25 mg/kg) with the second PET scan beginning 60 minutes post-tiagabine administration. Calculated tiagabine dose was rounded to the nearest even number to prevent tablet splitting (lowest dosage form is a 2 mg tablet).

The scanning protocol was identical for all scans. PET imaging was performed with the ECAT EXACT HR+ (Siemens/CTI, Knoxville, TN). A 10 min transmission scan was obtained prior to each radiotracer injection for attenuation correction of the emission data. [^11^C]Flumazenil was produced via a modification of published procedures [18]. Twenty mCi or less of high specific activity [^11^C]flumazenil was injected i.v. over 30 sec. Emission data were collected in the 3D mode for 90 min as 19 successive frames of increasing duration (4×15 s, 3×1 min, 3×2 min, 2×5 min, 7×10 min). Subjects were allowed to rest outside of the camera for 30–60 min between the two injections. Subjects remained in the Montefiore University Hospital Clinical and Translational Research Center overnight after the PET scans to monitor for any side effects of tiagabine administration. Adverse effects observed in this study included sedation (mild to moderate) and ataxia (mild) and resolved completely within 4 hours of the scan.

### Input function measurement

Following radiotracer injection, arterial samples were collected manually approximately every 6 s for the first two min and thereafter at longer intervals. A total of 35 samples were obtained per scan. Following centrifugation (2 min at 12,500 rpm, Spectrafuge 16M), plasma was collected in 200 µL aliquots and activities were counted in a gamma counter (Packard Biosciences).

To determine the plasma activity representing unmetabolized parent compound, seven samples (collected at 2, 5, 15, 30, 45, 75 and 90 min) were further processed via aqueous/organic extraction [19] to measure the fractional concentrations of hydrophilic metabolites and unchanged (lipophilic) [^11^C]flumazenil. The seven measured, unmetabolized fractions were fitted to the sum of one exponential plus a constant and this function was used to interpolate values between the measurements.

The input function was calculated as the product of total counts and interpolated unmetabolized fraction at each time point. The measured input function values were fitted to a sum of three exponentials from the time of peak plasma activity and the fitted values were used as the input to the kinetic analysis. The clearance of the parent compound (L/h) was calculated as the ratio of the injected dose to the area under the curve of the input function [20].

For the determination of the plasma free fraction (*f*_P_), triplicate aliquots of plasma collected prior to injection were mixed with the radiotracer, pipetted into ultrafiltration units (Amicon Centrifree; Bedford, MA) and centrifuged at room temperature (30 min at 6000 rpm). At end of centrifugation, the plasma and ultrafiltrate activities were counted (Packard Biosciences), and *f*_P_ was calculated as the ratio of activity in the ultrafiltrate to total activity [21]. Triplicate aliquots of saline solution mixed with the radiotracer were also processed, to determine the filter retention of the free tracer.

### MRI acquisition and segmentation procedures

To provide an anatomical framework for analysis of the PET data, MRI scans were obtained using a 1.5 T GE Medical Systems (Milwaukee, WI) Signa Scanner. A 3D spoiled gradient recalled sequence was acquired in the coronal plane using parameters that were optimized for maximal contrast among gray matter, white matter, and CSF. The scalp and calvarium were removed from the SPGR MR images, to facilitate MR/PET coregistration, using a manual in-house stripping technique. MRI segmentation was performed using the FAST automated segmentation tool [22] implemented in the FMRIB Software Library, v4.0 [23].

### Image analysis

PET data were reconstructed using filtered back-projection (Fourier rebinning/2D backprojection, 3 mm Hann filter) and corrected for photon attenuation (^68^Ge/^68^Ga rods), scatter [24], and radioactive decay. Reconstructed image files were then processed with the image analysis software MEDx (Sensor Systems, Inc., Sterling, Virginia) with the PET-MR image alignment performed using the SPM2 package (www.fil.ion.ucl.ac.uk/spm). PET data were inspected for subject motion and inter-frame motion was corrected using the realign procedure within SPM2, if necessary.

Regions of interest (ROIs) were drawn on each individual's MRI according to criteria derived from brain atlases [25], [26] and applied to the coregistered dynamic PET data to generate regional time-activity curves. Three functionally-based cortical ROIs were obtained as weighted averages of component ROIs: Association Cortex (dorsolateral prefrontal, orbital frontal, medial prefrontal, anterior cingulate), Sensory Cortex (parietal, occipital), and the limbic Medial Temporal Lobe (MTL; amygdala, hippocampus, enterorrhinal ctx and parahippocampal gyrus).

For the neocortical regions, “large” regions were first drawn to delineate the boundaries of the ROIs. Within these regions, only the voxels classified as gray matter were used to measure the activity distribution. Sampled volumes of the neocortical regions (n = 6) were as follows: dorsolateral prefrontal cortex (DLPFC, 19390±2899 mm^3^), orbito-frontal cortex (OFC, 10260±2448 mm^3^), medial prefrontal cortex (MPFC, 5001±1660 mm^3^), anterior cingulate cortex (ACC, 2587±686 mm^3^), parietal cortex (PC, 74583±13081 mm^3^), and occipital cortex (OC, 49286±7651 mm^3^).

Because of the mixture of gray and white matter in the structures of the MTL, the segmentation-based approach was not used for the component ROIs, and the boundaries of these regions were identified by anatomical criteria. These regions (n = 4) included amygdala (AMY, 2410±404 mm^3^), hippocampus (HIP, 4729±877 mm^3^), entorhinal cortex (ENT 960±243 mm^3^), and parahippocampal gyrus (PHG, 6021±1382 mm^3^).

For bilateral regions, right and left values were averaged. The contribution of plasma total activity to the regional activity was calculated assuming a 5% blood volume in the regions of interest [27] and tissue activities were calculated as the total regional activities minus the plasma contribution.

### Derivation of distribution volumes

We denote here the outcome variables using the consensus nomenclature for in vivo imaging of reversibly binding radioligands [28]. Derivation of [^11^C]flumazenil regional tissue distribution volume (*V*_T_, mL g^−1^) was performed with kinetic modeling using the arterial input function and an unconstrained two tissue compartment model (2TC model). *V*_T_, which is equal to the ratio of tissue to plasma parent activity at equilibrium, was derived as K_1_/k_2_(1+k_3_/k_4_), where K_1_ (ml g^−1^ min^−1^) and k_2_ (min^−1^) are the unidirectional fractional rate constants governing the transfer into and out of the brain, respectively, and k_3_ (min^−1^) and k_4_ (min^−1^) are the unidirectional fractional rate constants governing the association and dissociation of [^11^C]flumazenil to and from the BZD-site, respectively [29], [30], [31]. Kinetic parameters were derived by nonlinear regression using a Levenberg-Marquart least-squares minimization procedure [32] implemented in MATLAB (The Math Works, Inc., South Natick, MA), as previously described [30]. Given the unequal sampling over time (increasing frame acquisition time from the beginning to the end of the study), the least-squares minimization procedure was weighted by the frame acquisition time.

In previous studies, including our own [7], the pons has been used as the region of reference as activity in this region has been reported to represent predominantly nonspecific binding [21], [31], [33]. However, postmortem studies [34], [35], [36] as well as previous receptor imaging studies [20], including unpublished imaging data from our lab in humans, have demonstrated significant (up to 60%) of the signal from the pons is due to specific binding. While in our previous study tiagabine administration did not alter the pons *V*_T_ (equilibrium nonspecific binding, *V*_ND_) since the current study compared two dose of tiagabine we elected to utilize the *V*_T_ as our main outcome measure. However, for the purpose of comparison, we include the [^11^C]flumazenil binding potential relative to the total plasma concentration of [^11^C]flumazenil (*BP*_P_, mL g^−1^) derived as the difference between *V*_T_ in the ROI and *V*_ND_ (pons *V*_T_). The relationship between *BP*_P_ and BDZ receptor parameters is given by *BP*_P_ = *f*_P_*B_max_/K_D_, where B_max_ is GABA-A BDZ-receptor density, 1/K_D_ is the in vivo affinity of [^11^C]flumazenil for the GABA-A BDZ-receptor and *f*_P_ is the fraction of the radiotracer unbound to protein in the plasma.

### Derivation of Affinity Shift via Linear Regression Analysis

Lassen et al [37] demonstrated the ability to estimate the fractional change in binding potential (ΔBP) as the slope of the linear regression of the difference in *V*_T_ across conditions vs. the baseline *V*_T_ for cases where only the distribution volume and not the binding potential can be measured. In this study the linear regression of (*V*_T baseline_ - *V*_T post tiagabine_) vs. *V*_T baseline_ results in a line with a slope of ΔBP and an x-intercept of *V*_ND_. Assuming the affinity shift secondary to increased GABA levels is the same across all regions the shift in affinity is equal to 1- ΔBP, or 1 – slope of the plot of (*V*_T baseline_ - *V*_T post tiagabine_) vs. *V*_T baseline_. In each subject the percent change in affinity as well as *V*_ND_ was determined via this method using the values of *V*_T_ from the ten ROIs examined in the study (DLPFC, OFC, MPFC, ACC, PC, OC, AMY, HIP, ENT, and PHG) pre- and post-tiagabine administration.

### Electrophysiology and Cognitive Task

In all subjects the electrophysiology study was performed approximately 1 week prior to the PET scans. The Preparing to Overcome Prepotency (POP) task is a cued stimulus-response reversal paradigm that requires increases in cognitive control to overcome prepotent response tendencies [38]. Trials proceeded in the following order: cue (a green or red square; 500 ms); delay period (1000 ms); probe (a white arrow pointing left or right; 1000 ms); and a variable inter-trial interval (1000–2000 ms). Cues indicated conditions requiring either low (green square) or high (red square) degrees of cognitive control. Over the delay period, subjects were required to maintain the trial-type information and prepare for a response to the upcoming probe. For low-control trials, subjects were required to respond in the direction of the arrow that followed (e.g., for a right-pointing arrow, press the right button); for the high-control trials, responses were required in the opposite direction (e.g., for a right-pointing arrow, press the left button). To reinforce the prepotency of the cue-probe mappings of the low-control trials, thereby increasing the control requirements during the high-control trials, 70% of the trials were low-control and the remaining 30% were high-control. Trial types were interleaved in pseudorandom order, with 8 blocks of 42 trials each.

During the POP task, EEG data were acquired using a 129 Ag-AgCl coated carbon fiber electrode Geodesic Sensor Net (EGI, Eugene, OR) with a sampling frequency of 250 Hz. Data were filtered on-line with a 0.1–100 Hz band pass hardware filter. Electrode impedances were kept below 50 kΩ. All channels were referenced to Cz. Epochs were defined as −400 to +1900 ms relative to the cue onset. Error trials and epochs containing artifacts were excluded (20 channels with amplitude range exceeding 200 µV within a segment and/or having 60 µV deviations between consecutive samples). Segments identified by these criteria were visually inspected prior to rejection. Blink and ECG artifacts were removed with ICA based detection and correction methods. Data were filtered off-line using an 1–100 Hz finite impulse response filter. The resulting data were submitted to final review using the above amplitude and gradient criteria and for 60 Hz line noise, with bad channel data being replaced by interpolation. Data were re-referenced to average reference [39]. Average segment counts for the high and low control conditions were 220 and 80, respectively.

Time-frequency analyses were carried out using Brain Vision Analyzer (Brain Products GmbH, Munich, Germany). The data were transformed using complex Morlet wavelet transforms, defined by mo(x) = c⋅exp(−x^2^/2)⋅exp(iω_0_x), with c = 7, using 20 frequency steps spanning 14–80 Hz. Wavelet transformed data were baseline corrected to a −300 to −100 ms pre-cue interval.

EEG analysis involved identifying frontal areas of peak induced (i.e., not time locked to stimulus) gamma activity differences between the high and low control conditions, with a focus on the delay period (500–1500 ms relative to cue onset) during which induced frontal gamma activity has been shown to modulate in accordance with cognitive control demands [38]. Activity from one representative electrode from each of the left and right frontal areas, respectively, was averaged to derive a spatially averaged measure for comparison to the PET data. The peak activity was in the gamma sub-band with central frequency 42 Hz in a left frontal region consisting of adjacent electrode locations E23, E24 and E27 (approximately AF3 and AF7, respectively, in the 10-10 system). This procedure resulted in one summary measure of frontal gamma activity for each subject, which were then compared to the individual measurements of tiagabine induced increase in [^11^C]flumazenil binding by PET. Note that an experimenter blind to the PET data performed the determinations of these EEG measures of frontal gamma.

### Statistical analysis

Between scan comparisons were assessed with a paired, two-tailed t-test with a significance level of 0.05. Baseline and post-tiagabine K_1_ and *V*_T_ for the three functional cortical regions and *V*_ND_ (pons *V*_T_) were compared using a two-tailed, paired t-test, with an uncorrected probability value of 0.05 selected as the significance level. For the analysis of the tiagabine-induced change in *V*_T_ in the component ROIs (n = 10) a univariate repeated-measures analysis of variance (RM ANOVA) with brain regions as the within-scan factor and condition (baseline or post-tiagabine) as the between-scan factor was used. Paired t-tests were performed, when appropriate, to determine which regions accounted for significant effects observed in the RM ANOVA. The relationship between the PET scan outcome measures and the measurement of gamma-band power were analyzed with the Pearson product moment correlation coefficient after first confirming normal distribution of the data using the Kolmogorov-Smirnov test.

## Results

### PET scan parameters

No difference was observed in the injected dose, specific activity or injected mass of [^11^C]flumazenil between the baseline and post-tiagabine scan in either group (Table 1). Tiagabine administration did not affect the plasma clearance of [^11^C]flumazenil, [^11^C]flumazenil plasma free fraction (*f*_P_) or the distribution volume (*V*_T_) of [^11^C]flumazenil in the pons (Table 1).

**Table 1:** Demographic and scan data.

	Dose Group I			Dose Group II		
Parameter	Baseline	Post-tiagabine	p	Baseline	Post-tiagabine	p
N	9	9	-	9	9	-
Age	23.7±2.5	-	-	30.6±10.9	-	-
Gender	4M/5F	-	-	5M/4F	-	-
Ethnicity	1AA/7C/1H	-	-	3AA/6C	-	-
Tiagabine						
Dose (mg/kg)	-	0.14±0.01	-	-	0.24±0.01	-
Plasma level^1^ (ng/mL)	-	141±55	-	-	291±69	-
Injected dose (mCi)	19.9±2.0	20.4±1.8	0.45	20.9±0.8	21.0±1.0	0.72
SA (Ci/mmoles)	1963±757	2007±693	0.89	1837±1048	2254±2068	0.55
Injected Mass (ug)	3.4±0.9	3.5±1.6	0.79	4.6±3.0	4.7±3.9	0.86
Free Fraction (f_P_, %)	58.0%±7.6%	59.6%±6.1%	0.31	54.6%±7.8%	56.9%±5.2%	0.12
Clearance (L/h)	53±67	53±23	1.00	43±22	56±25	0.23
Pons *V*_T_ (or *V*_ND_ mL/g)	1.0±0.1	1.0±0.1	0.56	0.9±0.1	1.0±0.1	0.17

AA, African-American, AS, Asian, C, Caucasian. Significance level given is for a paired, two-tailed t-test.

1 Plasma level take at the time of the scan.

### Regional distribution volumes, *K*_1_ values and BZD receptor availability

**Dose Group I:** Administration of 0.15 mg/kg tiagabine did not result in a significant change in *V*_T_ in any of the large cortical regions (these and subsequent values noted as mean ± standard deviation, p values for two-tailed, paired t-test unless otherwise stated); Association Cortex 7.6±0.6 mL g^−1^ vs. 7.7±0.4 mL g^−1^ (p = 0.84), Sensory Cortex 7.5±0.5 mL g^−1^ vs. 7.3±1.0 mL g^−1^ (p = 0.64) and limbic Medial Temporal Lobe (MTL) 5.9±0.5 mL g^−1^ vs. 5.9±0.3 mL g^−1^ (p = 0.86). Examination of *V*_T_ across the component ROIs revealed significant regional effect (RM ANOVA F = 186.3, df = 9, 8, p<0.0001), no region by condition interaction (RM ANOVA F = 0.51, df = 9, 8, p = 0.84) and no significant difference across conditions (RM ANOVA F = 0.008, df = 1, 16, p = 0.93), see Table 2.

**Table 2:** Tiagabine-induced change in [11C]flumazenil *V*_T_ in control subjects.

	Dose Group I (0.15 mg/kg)					Dose Group II (0.25 mg/kg)				
Subdivision- Component ROIs	Baseline *V*_T_	Post-tiagabine *V*_T_	Δ*V*_T_(%)	d	p	Baseline *V*_T_	Post-tiagabine *V*_T_	Δ*V*_T_(%)	d	p
**Association Cortex**	**7.6±0.6**	**7.7±0.4**	**0.8±6.5**	**−0.07**	**0.84**	**6.8±0.8**	**7.3±0.4**	**9.3±11.4**	**−0.99**	**0.03**
-DLPFC	7.6±0.6	7.6±0.4	0.5±6.0	−0.03	0.92	6.7±0.8	7.2±0.4	9.5±11.2	−1.00	0.03
-Orbital Frt Ctx	7.5±0.7	7.5±0.6	0.4±7.8	−0.01	0.98	6.7±0.7	7.2±0.5	8.0±13.0	−0.82	0.11
-MPFC	8.0±0.6	8.1±0.5	1.0±7.0	−0.11	0.77	7.1±1.0	7.8±0.5	11.2±12.3	−0.92	0.02
-Ant. Cingulate Ctx	7.8±0.6	8.0±0.5	2.9±7.9	−0.38	0.36	7.2±0.8	7.7±0.5	7.5±11.3	−0.72	0.08
**Sensory Cortex**	**7.5±0.5**	**7.3±1.0**	**−1.9±14.9**	**0.26**	**0.64**	**6.7±0.8**	**7.3±0.5**	**9.7±10.8**	**−0.97**	**0.02**
-Parietal Ctx	7.3±0.4	7.4±0.4	0.8±8.1	−0.08	0.87	6.6±0.8	7.1±0.5	9.5±11.3	−0.88	0.03
-Occipital Ctx	7.7±0.6	7.4±1.5	−4.2±21.2	0.34	0.53	7.0±0.8	7.6±0.4	9.7±10.2	−1.06	0.02
**Medial Temporal Lobe**	**5.9±0.5**	**5.9±0.3**	**0.0±7.8**	**0.07**	**0.86**	**5.2±0.6**	**5.7±0.3**	**9.4±10.7**	**−1.02**	**0.03**
-Amygdala	5.9±0.6	5.8±0.4	−0.6±10.1	0.14	0.73	5.2±0.6	5.6±0.4	8.0±12.0	−0.79	0.11
-Hippocampus	6.0±0.5	5.9±0.3	−1.1±8.3	0.23	0.58	5.1±0.5	5.6±0.4	10.1±10.6	−1.15	0.02
-Entor. Ctx	5.6±0.4	5.6±0.3	0.9±7.9	−0.09	0.84	5.1±0.7	5.5±0.3	9.7±11.6	−0.83	0.03
-Parahippocampus	6.0±0.5	6.0±0.3	0.6±7.2	−0.03	0.93	5.4±0.7	5.8±0.3	9.4±10.6	−0.89	0.02

Values are Mean ± SD, in healthy controls (n = 9 per group); p is the significance level of the difference between the baseline and post-tiagabine scans in each group (paired t-test); d is the Cohen's effect size of this difference.

Similarly, neither *K*_1_ (Table 3) nor *BP*_P_ (Table 4) were changed with the 0.15 mg/kg dose of tiagabine. Examination of *K*_1_ revealed a significant regional effect (RM ANOVA F = 265.4, df = 9, 8, p<0.0001), no region by condition interaction (RM ANOVA F = 0.47, df = 9, 8, p = 0.86) and no difference across conditions (RM ANOVA F = 1.3, df = 1, 16, p = 0.27). Examination of *BP*_P_ revealed a significant regional effect (RM ANOVA F = 186.4, df = 9, 8, p<0.0001), no region by condition interaction (RM ANOVA F = 0.51, df = 9, 8, p = 0.84) and no difference across conditions (RM ANOVA F = 0.05, df = 1, 16, p = 0.83).

**Table 3:** Tiagabine-induced change in [11C]flumazenil *K*_1_ in control subjects.

	Dose Group I (0.15 mg/kg)				Dose Group II (0.25 mg/kg)			
Subdivision- Component ROIs	Baseline *K*_1_	Post-tiagabine *K*_1_	d	p	Baseline *K*_1_	Post-tiagabine *K*_1_	d	p
**Association Cortex**	**0.441±0.03**	**0.463±0.04**	**−0.66**	**0.18**	**0.405±0.06**	**0.421±0.05**	**−0.31**	**0.27**
-DLPFC	0.447±0.03	0.467±0.04	−0.57	0.22	0.407±0.06	0.424±0.05	−0.34	0.21
-Orbital Frt Ctx	0.429±0.03	0.452±0.04	−0.67	0.23	0.394±0.06	0.411±0.05	−0.36	0.22
-MPFC	0.452±0.04	0.477±0.04	−0.72	0.15	0.419±0.07	0.438±0.05	−0.32	0.14
-Ant. Cingulate Ctx	0.425±0.04	0.455±0.04	−0.77	0.08	0.408±0.06	0.434±0.06	−0.45	0.09
**Sensory Cortex**	**0.438±0.04**	**0.447±0.07**	**−0.17**	**0.73**	**0.412±0.05**	**0.425±0.05**	**−0.28**	**0.21**
-Parietal Ctx	0.443±0.04	0.468±0.04	−0.66	0.18	0.412±0.05	0.432±0.05	−0.40	0.08
-Occipital Ctx	0.429±0.05	0.428±0.10	0.02	0.96	0.408±0.05	0.419±0.04	−0.25	0.28
**Medial Temporal Lobe**	**0.308±0.03**	**0.332±0.04**	**−0.68**	**0.10**	**0.291±0.04**	**0.304±0.03**	**−0.40**	**0.17**
-Amygdala	0.316±0.04	0.331±0.03	−0.42	0.33	0.295±0.04	0.298±0.03	−0.09	0.75
-Hippocampus	0.310±0.03	0.332±0.04	−0.67	0.06	0.287±0.03	0.309±0.02	−0.78	0.03
-Entor. Ctx	0.270±0.04	0.294±0.03	−0.72	0.07	0.264±0.04	0.280±0.03	−0.50	0.16
-Parahippocampus	0.308±0.03	0.331±0.04	−0.63	0.10	0.293±0.04	0.311±0.03	−0.52	0.06

Values are Mean ± SD, in healthy controls (n = 9 per group); p is the significance level of the difference between the baseline and post-tiagabine scans in each group (paired t-test); d is the Cohen's effect size of this difference.

**Table 4:** Tiagabine-induced change in [11C]flumazenil *BP*_P_ in control subjects.

	Dose Group I (0.15 mg/kg)					Dose Group II (0.25 mg/kg)				
Subdivision- Component ROIs	Baseline *BP*_P_	Post-tiagabine *BP*_P_	Δ*BP*_P_(%)	d	p	Baseline *BP*_P_	Post-tiagabine *BP*_P_	Δ*BP*_P_(%)	d	p
**Association Cortex**	**6.6±0.6**	**6.7±0.4**	**0.5±7.0**	**−0.02**	**0.95**	**5.9±0.7**	**6.4±0.4**	**9.5±12.7**	**−0.91**	**0.05**
-DLPFC	6.6±0.6	6.6±0.4	0.2±6.2	0.02	0.94	5.8±0.7	6.3±0.4	9.7±12.6	−0.91	0.05
-Orbital Frt Ctx	6.5±0.6	6.5±0.6	0.1±8.6	0.04	0.91	5.8±0.7	6.2±0.5	8.0±14.9	−0.70	0.17
-MPFC	7.0±0.5	7.1±0.5	0.7±7.6	−0.06	0.87	6.2±0.9	6.8±0.5	11.6±13.1	−0.87	0.03
-Ant. Cingulate Ctx	6.8±0.6	7.0±0.4	3.0±8.8	−0.34	0.41	6.3±0.8	6.7±0.6	7.4±12.8	−0.62	0.14
**Sensory Cortex**	**6.5±0.5**	**6.3±0.9**	**−2.5±17.1**	**0.30**	**0.60**	**5.8±0.7**	**6.3±0.5**	**10.0±11.8**	**−0.90**	**0.03**
-Parietal Ctx	6.3±0.4	6.4±0.4	0.5±9.3	−0.02	0.97	5.7±0.7	6.2±0.5	9.7±12.5	−0.81	0.04
-Occipital Ctx	6.7±0.6	6.3±1.5	−5.0±24.2	0.37	0.50	6.1±0.7	6.6±0.4	9.9±10.9	−0.99	0.02
**Medial Temporal Lobe**	**4.9±0.5**	**4.9±0.3**	**−0.5±8.2**	**0.14**	**0.71**	**4.3±0.6**	**4.7±0.3**	**9.7±11.6**	**−0.91**	**0.04**
-Amygdala	4.9±0.6	4.8±0.5	−1.3±10.5	0.19	0.61	4.4±0.5	4.6±0.3	8.0±13.0	−0.69	0.15
-Hippocampus	5.0±0.5	4.9±0.4	−1.8±8.7	0.28	0.43	4.2±0.5	4.6±0.3	10.6±11.2	−1.04	0.02
-Entor. Ctx	4.6±0.4	4.6±0.3	0.5±8.8	−0.01	0.98	4.2±0.7	4.6±0.3	10.3±13.5	−0.69	0.06
-Parahippocampus	5.0±0.5	5.0±0.4	0.3±7.9	0.03	0.93	4.5±0.6	4.8±0.3	9.7±11.7	−0.78	0.03

Values are Mean ± SD, in healthy controls (n = 9 per group); p is the significance level of the difference between the baseline and post-tiagabine scans in each group (paired t-test); d is the Cohen's effect size of this difference.

**Dose Group II:** After the administration of 0.25 mg/kg tiagabine *V*_T_ increased significantly in the large cortical regions; Association Cortex 6.8±0.8 mL g^−1^ vs. 7.3±0.4 mL g^−1^ (p = 0.03), Sensory Cortex 6.7±0.8 mL g^−1^ vs. 7.3±0.5 mL g^−1^ (p = 0.02) and limbic Medial Temporal Lobe (MTL) 5.2±0.6 mL g^−1^ vs. 5.7±0.3 mL g^−1^ (p = 0.03). Examination of *V*_T_ across the component ROIs revealed a significant regional effect (RM ANOVA F = 118.9, df = 9, 8, p<0.0001), no region by condition interaction (RM ANOVA F = 0.42, df = 9, 8, p = 0.42) and a trend-level difference across conditions (RM ANOVA F = 4.0, df = 1, 16, p = 0.06). On a region-by-region basis, significant increases in all regions were seen post-tiagabine (Table 2) with the exception of the orbital prefrontal cortex (ORB, p = 0.11), anterior cingulate cortex (ACC, p = 0.08) and the amygdala (AMY, p = 0.11). No significant correlations between age or tiagabine plasma concentration and *V*_T_ increase were noted.

As observed in the lower dose group *K*_1_ (Table 3) did not change significantly with the administration of 0.25 mg/kg of tiagabine; a significant regional effect (RM ANOVA F = 249.7, df = 9, 8, p<0.0001), no region by condition interaction (RM ANOVA F = 0.57, df = 9, 8, p = 0.82) and no difference across conditions (RM ANOVA F = 0.66, df = 1, 16, p = 0.43) were observed.

However, as opposed to the lower dose level, *BP*_P_ increased significantly in the large cortical regions with 0.25 mg/kg of tiagabine (Table 4); Association Cortex 5.9±0.7 mL g^−1^ vs. 6.4±0.4 mL g^−1^ (p = 0.05), Sensory Cortex 5.8±0.7 mL g^−1^ vs. 6.3±0.5 mL g^−1^ (p = 0.03) and limbic Medial Temporal Lobe (MTL) 4.3±0.6 mL g^−1^ vs. 4.7±0.3 mL g^−1^ (p = 0.04). Examination of *BP*_P_ across the component ROIs revealed a significant regional effect (RM ANOVA F = 118.9, df = 9, 8, p<0.0001), no region by condition interaction (RM ANOVA F = 0.42, df = 9, 8, p = 0.89) and a trend-level difference across conditions (RM ANOVA F = 3.31, df = 1, 16, p = 0.09).

### Linear Regression Analysis

In Dose Group I, given the lack of change in *V*_T_, the linear regression analysis did not result in meaningful data. In Dose Group II the average slope was −0.27±2.84 and the average x-intercept was 1.53±7.14 (n = 9 subjects). The affinity shift, calculated as 1 – slope, was 1.27±2.84, in other terms, on average, a 27% increase in affinity was observed across subjects in this dose group.

### Electroencephalogram (EEG) induced gamma-band oscillations

**Figure 1:**
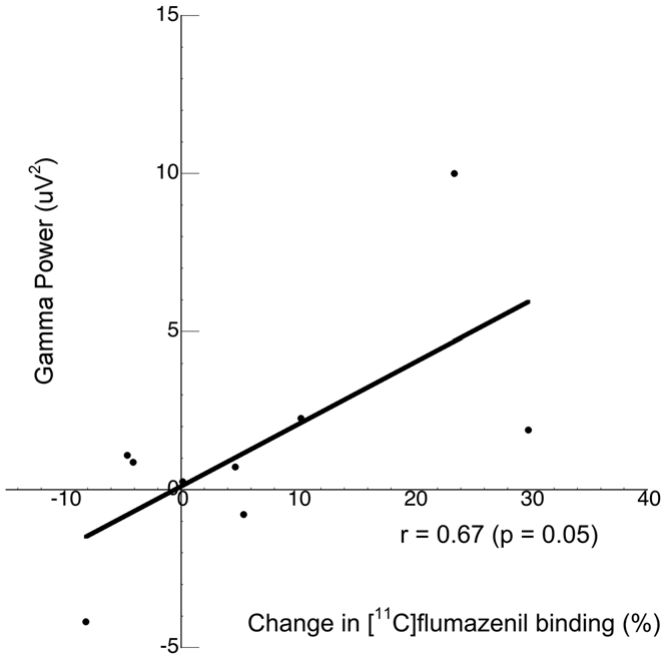
The ability to increase GABA levels in the orbitofrontal cortex, measured as the change in [^11^C]flumazenil binding in response to GAT1 blockade, predicts (r = 0.67, p = 0.05) the ability to entrain cortical networks, measured via EEG gamma oscillations.

To confirm our previous results which indicated that individuals with greater capacity to increase extracellular GABA levels post-tiagabine (a “GABA reserve”) would exhibit enhanced frontal gamma-band oscillatory activity in the context of a task that taps cognitive control processes [7], all subjects underwent EEG measurement of frontal lobe gamma-band oscillations during the Preparing to Overcome Prepotency (POP) task [38]. Frontal cortical gamma-band power was measured in each individual during the delay period. Given that no change in [^11^C]flumazenil binding was noted in Dose Group I, it would not be expected to note an association between change in [^11^C]flumazenil binding and gamma band power; in fact this was confirmed (r = 0.12, p = 0.76). For Group II the association between gamma-band power and the ability to increase extracellular GABA levels was significant in the orbital frontal cortex (r = 0.67, p = 0.05; Figure 1) but in none of the other regions examined, although the directionality was the same across all regions. No relationship was observed between behavioral performance on the POP task and gamma-band power or change in [^11^C]flumazenil binding as all individuals performed at a high level on the task.

## Discussion

The results of this study confirm our previous finding that acute increases in extracellular cortical GABA can be detected as an increase in binding of the BDZ-site specific radiotracer, [^11^C]flumazenil. Moreover, these data indicate a dose-response relationship in the magnitude of the increase in [^11^C]flumazenil binding and provide an estimate of the change in affinity at the BZD-site (27% increase) resulting in the increased binding. A critical assumption of this study is that systemic administration of tiagabine results in an acute, dose-dependent, increase in extracellular GABA. Tiagabine is a highly selective GAT1 blocker, without significant affinity for other receptors which, in humans, exhibits linear pharmacokinetics over the dose range of 2–24 mg with high oral bioavailability (90%) and peak plasma levels at approximately 1 hr post-dose [40]. In humans, tiagabine dose has been correlated to changes in brain GABA levels by the observation that there is a significant relationship between dose and reduction in seizure frequency [41] and through a microdialysis study in a single patient which found a 50% increase in hippocampal GABA levels with a 16 mg oral dose, occurring 1 hr post-dose and sustained for several hours [42]. Rodent microdialysis studies have shown that ip injection of tiagabine (11.5 and 21 mg/kg) increases extracellular GABA concentration ∼200%–400% over basal levels in a dose-dependent manner, peaking 40–60 minutes following injection [43], [44] and after administration of tiagabine (1 mg/kg iv), a 2-fold increase in extracellular GABA levels was observed, reaching a maximum approximately 70 minutes post-tiagabine in vervet monkeys [45]. Taken together these preclinical studies support the assumption that extracellular GABA levels increase in a dose-dependent manner with tiagabine.

The principle underlying the hypothesis of a ‘GABA-shift’ is the enhancement in BDZ-receptor affinity for BDZ-site substrates resulting from increased GABA [9], [10]. It is widely accepted that BDZs potentiate the effects of GABA at the GABA-A receptor and the reverse is true, increased GABA levels potentiate the binding of BDZs to the GABA-A receptor in a dose-dependent manner [9]. Some in vitro studies exploring this phenomenon indicate that it is specific to agonist drugs [46] while other in vitro studies were not able to demonstrate the GABA-shift, even with BDZ agonists [47]. Two preclinical studies have examined the effects of increasing GABA and GABAergic drugs on flumazenil binding in intact animals and both found results consistent with our findings. The first [48] demonstrated enhanced [^3^H]flumazenil binding in vivo when mice were treated with either progabide (a GABA analog and agonist at the GABA-A receptor) or valproate, which increases brain GABA levels [49]. The second [8] measured the effects of three GABAergic drugs, aminooxyacetic acid (inhibitor of GABA transaminase, the enzyme that metabolizes GABA), gamma-vinyl GABA (GVG, irreversible GABA transaminase inhibitor) and valproate on [^3^H]flumazenil binding. All three drugs resulted in acute increases in cortical GABA concentrations and specific binding of [^3^H]flumazenil increased acutely across all brain regions, with all three drugs, with no change in nonspecific binding. These results, as well as those observed in our studies, suggest that while flumazenil acts as a benzodiazepine site antagonist in most settings it may also behave as a high affinity partial agonist with weak efficacy in others. This hypothesis is consistent with studies showing flumazenil to be anxiolytic in some rodent models of anxiety [50] as well as in normals in stressful (public speaking) settings but not at baseline [51], to reduced benzodiazepine withdrawal symptoms in dependent subjects [52] and to enhance the GABA-A receptor mediated currents evoked by GABA [53].

The strengths of the present study include measurement of the arterial input function, allowing for the assessment of the effects of tiagabine on *V*_ND_ and f_P_. While the absence of change in these variables post-tiagabine validates the use of either *BP*_P_ or *BP*_ND_ as an outcome measure, we chose to use *V*_T_, derived via 2TC modeling, as our primary outcome measure. Our rationale for selecting *V*_T_ as the outcome measure in this study was the postmortem studies [34], [35], [36], previous receptor imaging study [20] and unpublished data from our lab, all of which demonstrate a significant degree of specific binding in the pons (up to 60%). We were concerned that differential effects of elevated GABA levels on [^11^C]flumazenil specific binding in the pons would impact the comparison across tiagabine dose groups. Since we anticipated a greater increase in [^11^C]flumazenil binding in the high dose group, specific binding within the pons would impact either *BP*_P_ or *BP*_ND_ to a greater degree in this group relative to the low dose group potentially obscuring group differences in tiagabine induced change in [^11^C]flumazenil binding, despite the fact that, on average, no changes were seen in the pons *V*_T_ post tiagabine in either group. In fact, this is what we observed; as pons *V*_T_ is progressively included in the outcome measure there is a loss of significant differences likely secondary to elevations in pons *V*_T_ with tiagabine administration. Examination of *V*_T_ (Table 2), BP_P_ (Table 4) and BP_ND_ (data not shown) in the high dose group demonstrates a significant change in *V*_T_, a trend-level change in BP_P_, and no change in BP_ND_.

In our previously published study we noted a relatively high variability in the percent change in [^11^C]flumazenil binding across subjects. One factor we postulated may be involved in the variability was the timing of the PET scan relative to the administration of tiagabine [6]. In our initial study, we timed the PET scan such that scanning commenced at 30 min post dose to ensure the measurement occurred during the acute increase in GABA coinciding with the reported Tmax of the plasma concentration of tiagabine (45–60 min); however, microdialysis studies indicate GABA increases are sustained for several hours after oral administration of tiagabine. In the current study we increased the time between tiagabine administration and the start of PET scanning from 30 to 60 min, allowing for more consistent absorption of tiagabine across subjects. In addition we based the tiagabine dose on subject weight as opposed to using a standard, single, dose for each subject (as we did in our first study). Despite these changes in study design the variability in the percent change in [^11^C]flumazenil binding was not significantly altered nor did we detect a correlation between tiagabine plasma level and change in [^11^C]flumazenil binding (data not shown). In our first study we noted a significant increase in *V*_T_ across the ROIs used in this study (average increase in *V*_T_ of 13.7%±15.9%). This increase is comparable, albeit numerically larger, to that observed in Dose Group II, where we noted an average increase in *V*_T_ of 9.3%±10.9% across the 10 component ROIs listed in Table 2. Detecting differences between individuals with a psychiatric disorder and healthy controls may be challenging with this level of variability in the measurement; however, comparing the two groups in this study we noted the increase in [^11^C]flumazenil *V*_T_ to be greater for Group II vs. Group I at a trend level (p = 0.06, RM ANOVA). In other words, relatively large between-dose differences can be detected with the current method (the average increase in *V*_T_ for Dose Group I was 0.12%±9.7%) but more subtle differences in GABA availability may be difficult to detect without improvements in the methods to reduce the variability.

The goal of the present study was to validate our previous findings by demonstrating that the magnitude of increase in [^11^C]flumazenil binding observed with PET is directly correlated with the degree of increase in extracellular GABA. We utilized increased tiagabine dose as a proxy for increased extracellular GABA levels since the only direct method to examine this hypothesis would be combined PET/microdialysis studies in nonhuman primates, which are confounded by the necessary anesthesia. Our results provide additional evidence supporting the idea that the GABA-shift phenomenon can be observed in vivo, using PET, in humans. The ability to measure in vivo changes in extracellular GABA levels provides a unique opportunity to explore the role of GABA in certain brain processes. Studies with nonhuman primates demonstrate that the disruption of GABA transmission in the DLFPC impairs working memory [54]. In humans, DLPFC gamma oscillations normally increase with working memory load [55], a phenomena which is impaired in subjects with working memory deficits [38]. In addition to playing a role in working memory [5], gamma synchrony appears to be associated with other higher cognitive processes such as associative learning [4]. Although the specific role of GABA transmission in working memory is still under investigation, the synchronization of pyramidal cell firing by networks of fast-spiking, parvalbumin-containing GABA neurons gives rise to oscillatory activity in the gamma range [56]. While not powered to examine this issue specifically, in the current study, as in our previous one, we observed a relationship between the change in [^11^C]flumazenil binding (in dose Group II) and the ability to entrain oscillatory activity in the gamma frequency during a cognitive control task. While in this study we only observed this relationship with one ROI, this provides further direct support for the hypothesis that GABA neurotransmission is linked to the synchronization of cortical neuronal activity in humans. Continued refinement and validation of the PET methodology described in this study is necessary for it to consistently provide the ability to measure changes in GABA levels in vivo; however, it provides a unique method to explore differences between control and patient populations in the degree of extracellular GABA increase in response to a standardized level of GAT1 blockade.
